# Case Reports of Visceral Leishmaniasis-Associated Hemophagocytic Lymphohistiocytosis in Adults: A Complex Immune Phenomenon

**DOI:** 10.3390/reports9010029

**Published:** 2026-01-20

**Authors:** Touba Bougiouklou, Vasileios Petrakis, Ioulia Dragoumani, Evanthia Gouveri, Dimitrios Papazoglou

**Affiliations:** 2nd University Department of Internal Medicine, University General Hospital Alexandroupolis, Democritus University of Thrace, 68100 Alexandroupolis, Greece; toubabougiouklou@gmail.com (T.B.); juliadragoumani@gmail.com (I.D.); vangouv@yahoo.gr (E.G.); dpapazog@med.duth.gr (D.P.)

**Keywords:** visceral leishmaniasis, hemophagocytic lymphohistiocytosis, *Leishmania infantum*, adult HLH, hyperferritinemia, pancytopenia

## Abstract

**Background**: Visceral Leishmaniasis (VL), a severe systemic parasitic disease caused by *Leishmania* species, can be complicated by secondary Hemophagocytic Lymphohistiocytosis (HLH). HLH is a life-threatening hyperinflammatory syndrome characterized by excessive immune activation that results in multiorgan dysfunction. The co-occurrence of VL and HLH in adults is a rare but critical diagnostic and therapeutic challenge, often leading to fatal outcomes if treatment is delayed. **Case Presentation**: We present two cases of adult males (60 and 72 years old) from Greece, an endemic area for *L. infantum*, who presented with prolonged fever, pancytopenia, hepatosplenomegaly, and impaired liver function. Both patients exhibited extremely elevated ferritin (all > 2000 ng/mL and one > 20,000 ng/mL) and hypertriglyceridemia, fulfilling key laboratory criteria for HLH. Diagnosis was confirmed by the visualization of *Leishmania* amastigotes in bone marrow aspirates, which also demonstrated features of hemophagocytosis. Case 1, critically ill with acute kidney injury and coagulopathy, required combined treatment with liposomal Amphotericin B and immunoglobulin therapy for HLH. Case 2, who showed rapid and “spectacular improvement” solely after receiving liposomal Amphotericin B, did not require HLH-specific immunosuppression. **Conclusions**: VL-associated HLH should be considered in adult patients presenting with complex systemic inflammation, fever, and cytopenias, particularly in endemic settings. Our cases illustrate that the prompt initiation of anti-leishmanial therapy with liposomal Amphotericin B can be sufficient to reverse the HLH syndrome by eliminating the infectious trigger. However, intensive immunomodulation may be necessary in patients presenting with critical multi-organ failure.

## 1. Introduction and Clinical Significance

Leishmaniasis represents a spectrum of vector-borne diseases caused by obligate intracellular protozoa belonging to the genus *Leishmania* [1]. Transmission occurs through the bite of an infected female phlebotomine sandfly, and the parasites primarily target the host’s reticuloendothelial system [1]. The disease manifests in three principal forms: cutaneous leishmaniasis (CL), mucosal leishmaniasis (ML), and the most severe systemic form, visceral leishmaniasis (VL) [1]. Visceral leishmaniasis, also known as “kala-azar” or “black fever,” is characterized by irregular bouts of fever, pronounced hepatomegaly and splenomegaly, pancytopenia, and constitutional symptoms such as weight loss and night sweats [2]. The diagnosis of VL can be extremely complicated, particularly in non-endemic regions or when presenting atypically [3]. Globally, an estimated 30,000 cases of VL occur annually [4]. In 2024, about 85% of global VL cases were reported from seven countries: Brazil, Ethiopia, India, Kenya, Somalia, South Sudan and Sudan [4]. The incubation period is highly variable, ranging from a few weeks to several years, and initial infection can be asymptomatic or present with a subacute onset of symptoms [2,3]. Without timely and appropriate treatment, VL carries a fatality rate that can approach 100% within two years [1,5].

The critical diagnostic gap in Visceral Leishmaniasis (VL) lies in its ability to trigger Hemophagocytic Lymphohistiocytosis (HLH)—a hyperinflammatory ‘cytokine storm’ that is frequently misdiagnosed as refractory bacterial sepsis or hematologic malignancy, leading to fatal delays in targeted therapy [6]. While VL is a well-known parasitic infection, its progression to secondary HLH in adults represents a high-stakes clinical emergency where the window for life-saving intervention is narrow [6]. Secondary hemophagocytic lymphohistiocytosis (HLH) could be critical and often fatal complication arising from delayed or complicated VL [6]. HLH is a severe, life-threatening hyperinflammatory syndrome resulting from excessive, uncontrolled activation of the immune system, characterized by unchecked proliferation of benign histiocytes and T-lymphocytes, leading to massive cytokine release [7]. This cytokine storm, termed “cytokine-driven tissue damage,” may rapidly progress to multi-organ failure and death if the underlying trigger is not addressed [8]. HLH can be triggered by various conditions, including infections, malignancy, or autoimmune disorders, in both immunocompromised and immunocompetent patients [9,10,11]. Identifying the underlying condition is vital, as targeted treatment often leads to clinical improvement of the HLH features, potentially avoiding more toxic, blanket immunosuppressive therapies [12,13,14].

VL and HLH share significant clinical overlaps, including fever, hepatosplenomegaly, and pancytopenia [2,7]. However, HLH is further delineated by specific laboratory markers such as hypertriglyceridemia, hypofibrinogenemia, significantly elevated ferritin levels (typically >500 ng/dL), and low or absent natural killer (NK) cell activity [15]. While HLH is primarily recognized as a pediatric syndrome, it is increasingly reported in adults, where a slight male predisposition has been noted [16]. The therapeutic approach for HLH traditionally relies on immunosuppressive and cytotoxic agents [11]. However, these interventions are frequently contraindicated in the setting of severe infections, necessitating a definitive diagnosis prior to initiation [11]. In cases of HLH associated with infections severe immunosuppression without an adequate antimicrobial agent could be devastating [11]. Targeted antileishmanial therapy is the cornerstone of managing VL-associated HLH [11]. In cases where the cytokine release syndrome has been progressed, the addition of low-dose corticosteroids and immunoglobulins to the treatment could be beneficial to cease the cytokine storm [11].

The co-occurrence of VL and HLH, particularly in the adult population, is rare and poses a profound diagnostic and therapeutic challenge. Prompt recognition is crucial given the high potential for a fatal outcome. We present two adult case reports of VL, without travel history, complicated by secondary HLH from Greece, where *L. infantum* is the main endemic species, aiming to reinforce the clinical index of suspicion for this complex immune phenomenon.

## 2. Case Presentation

### 2.1. Case 1

A 60-year-old male was admitted to the Department of Internal Medicine with a primary complaint of persistent fever spanning 20 days, accompanied by anorexia, generalized weakness, weight loss, and progressive jaundice. His past medical history was significant for a stroke, percutaneous coronary intervention (PCI) one year prior, chronic kidney disease (CKD) with a baseline creatinine of 1.4 mg/dL, hypertension, dyslipidemia, chronic obstructive pulmonary disease (COPD), and chronic systemic alcohol consumption initiated at age 20.

Upon physical examination, the patient was febrile, severely pale, and jaundiced. Abdominal palpation confirmed hepatosplenomegaly. Initial laboratory investigations revealed profound haematological abnormalities, specifically severe anaemia (Hemoglobin [Hb]: 6.8 g/dL, Hematocrit [Hct]: 20%, Mean Corpuscular Volume [MCV]: 78.7 fl) and severe thrombocytopenia (Platelets [PLT]: 34,000/μL), with normal total white blood cell (WBC) counts. There was no evidence of active bleeding or haemolysis. Inflammatory markers were highly elevated (C-reactive protein [CRP]: 16.81 mg/dL). Biochemical tests indicated acute kidney injury (Cr: 4.4 mg/dL) and significant hepatic dysfunction (AST: 158 U/L, ALT: 60 U/L, total bilirubin: 10.08 mg/dL, direct bilirubin: 6.66 mg/dL, ALP: 148 U/L, γ-GT:180 U/L). Coagulation parameters were prolonged (INR: 2.41, APTT: 51.8 s). Furthermore, markers highly suggestive of HLH were identified: ferritin was extremely elevated (>2000 ng/mL) and hypertriglyceridemia was noted (839 mg/dL).

Blood and urine cultures were obtained, and empirical broad-spectrum antibiotic treatment, consisting of Ceftazidime/Avibactam and Daptomycin, was initiated. The diagnostic workup included a CT scan of the chest and abdomen, which confirmed the hepatosplenomegaly and revealed small splenic abscesses, two hepatic cysts, and a few enlarged intra-abdominal lymph nodes. Extensive microbiological examination, including serological tests for *Leishmania*, was performed. Immunoglobulin M (IgM) antibodies for Leishmaniasis were found to be positive. While Staphylococcus haemolyticus was isolated from the tip of the central venous catheter (CVC), the organism was sensitive to the antibiotics already received. To confirm the parasitic diagnosis, a bone marrow aspiration (BMA) was performed. Microscopic examination of the BMA confirmed the presence of *Leishmania* amastigotes, thereby establishing the diagnosis of VL. The BMA also concurrently confirmed the diagnosis of HLH. Furthermore, molecular analysis via polymerase chain reaction (PCR) was performed and infection by *Leishmania infantum* was confirmed. Following confirmation, targeted therapy was initiated. Liposomal Amphotericin B was administered for VL at a dose of 3 mg/kg/day administered on days 1–5, 10, and 21, and immunoglobulin therapy was added to manage the HLH component, given the patient’s critical state involving acute kidney injury (AKI) and severe coagulopathy. The patient demonstrated gradual clinical and laboratory improvement, achieving stability, and was subsequently discharged after 21 days of hospitalization.

### 2.2. Case 2

A 72-year-old male was admitted due to persistent, high-grade fever (up to 38.5 °C) lasting one month. His medical history included benign prostatic hyperplasia (BPH), hypertension, dyslipidaemia, and hyperuricemia. The patient reported having consulted multiple physicians and received various antibiotic treatments without any clinical response or improvement. He also reported anorexia, weakness, and weight loss, but lacked other systemic symptoms. Physical examination revealed only an atrial valve systolic murmur. Subsequent heart ultrasound identified aortic valve stenosis and mild aortic valve insufficiency but ruled out infectious endocarditis. Abdominal examination was unremarkable for organomegaly. Laboratory analysis showed mild anaemia (Hb: 10.3 g/dL) and thrombocytopenia (PLT: 85,000/μL), with normal WBC counts. Similarly to Case 1, inflammatory markers were high (CRP: 13.76 mg/dL), and liver enzyme levels were elevated (AST: 236 U/L, ALT: 124 U/L). Crucially, HLH markers were severely elevated: ferritin exceeded 20,000 ng/mL and hypertriglyceridemia was present (307 mg/dL).

Empirical antibiotic treatment with Piperacillin/Tazobactam and Doxycycline was initiated after blood cultures were received. A CT scan of the chest and abdomen was performed, which revealed mild splenomegaly with multiple small splenic abscesses and a few hepatic microcysts. Serological testing indicated positive IgM antibodies for leishmaniasis, while blood and urine cultures remained negative. Bone marrow aspiration and biopsy were performed. The BMA confirmed the presence of the *Leishmania* parasite, confirming the VL diagnosis. The bone marrow biopsy concurrently diagnosed HLH. Molecular analysis via PCR was performed and infection by *Leishmania infantum* was confirmed. Liposomal Amphotericin B was administered for VL at a dose of 3 mg/kg/day administered on days 1–5, 10, and 21. The patient demonstrated a spectacular and rapid clinical and laboratory improvement following this treatment. The diagnosis of HLH was confirmed after the patient’s discharge from the hospital; thus, no specific HLH treatment beyond the antileishmanial therapy was administered.

**Figure 1:**
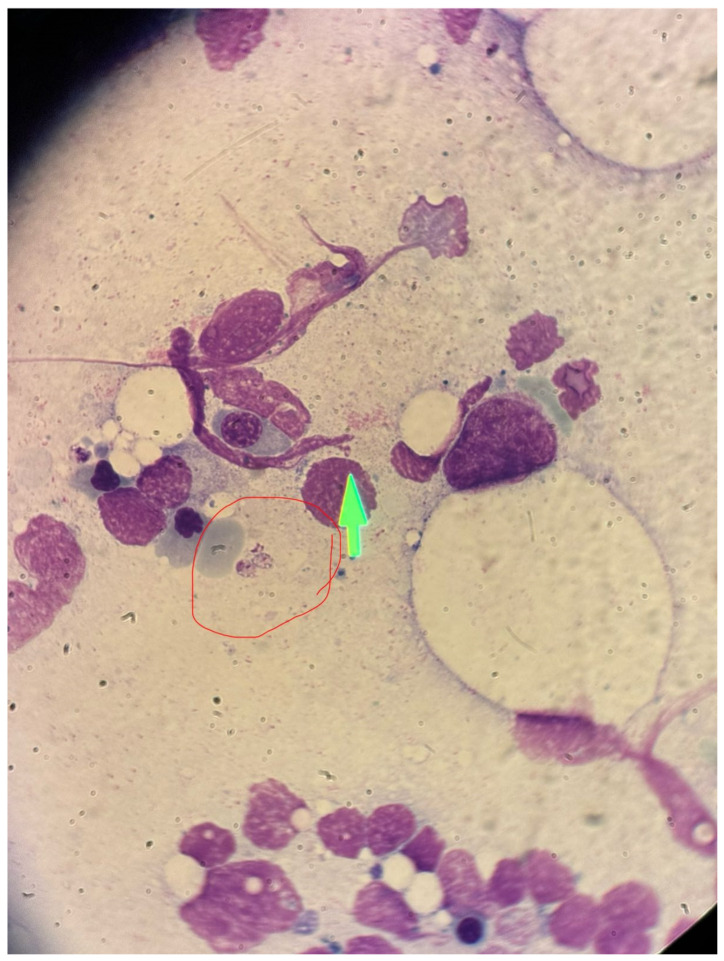
Photomicrograph of the bone marrow aspirate smear of Case 1. Red circle and green arrow are showing the presence of Leishmania amastigotes within a macrophage (Leishman-Donovan bodies). Magnification, 1000×.

The shared and distinct laboratory findings for both patients are summarized in Table 1. The definitive diagnosis in both cases relied on the visualization of *Leishmania* amastigotes within macrophages in the bone marrow aspirates. The bone marrow examination was instrumental in confirming not only the parasitic infection but also the concurrent features of HLH. Figure 1 provides a photomicrograph illustrating the presence of *Leishmania* amastigotes in the bone marrow aspirate of patient of Case 1.

## 3. Discussion

In Greece, *Leishmania infantum*, which causes VL, is the main endemic species [17]. Historically, the disease has a low average annual impact in the country (0.5 cases per 100,000 population during 2004–2022), with the 0–4 age group traditionally being the most frequently affected demographic [17]. Our two patients, both middle-aged males with multiple pre-existing health conditions, deviate from this expected paediatric predominance. This highlights the importance of considering VL in adult patients presenting with the classic triad of fever, hepatosplenomegaly, and pancytopenia, especially those with comorbidities that may predispose them to severe infection or who reside in or have travelled to endemic zones. These 2 patients were permanent residents of a peri-urban area in Northeastern Greece with no history of recent travel abroad, suggesting autochthonous acquisition of the infection in an endemic setting. The deviation from the expected pediatric predominance in our series may be justified by the patients’ long-term residence in endemic areas where sandfly vectors are prevalent in both rural and peri-urban environments. In these settings, cumulative environmental exposure over decades, coupled with age-acquired comorbidities—such as the cardiovascular and renal conditions noted in our patients—likely predisposes adults to the reactivation or primary clinical manifestation of *L. infantum*. This suggests that in the Mediterranean basin, the clinical profile of visceral leishmaniasis is evolving into an opportunistic or age-related pathology rather than a strictly pediatric one.

Both patients experienced a diagnostic lag, a phenomenon frequently observed even in endemic regions like Greece. This delay is often not due to a lack of regional awareness, but rather to the fact that VL is less commonly suspected in adult patients compared to children. Furthermore, the initial presentation of VL-associated HLH closely mimics other, more prevalent adult conditions such as bacterial sepsis, occult malignancy, or autoimmune disorders, which often dominate the initial differential diagnosis. The findings of splenomegaly with multiple small abscesses and liver cysts observed on CT scans in both patients are noteworthy atypical features that further complicated the initial diagnostic picture, necessitating an extensive workup to rule out other causes such as occult malignancy or deep fungal infection.

Hemophagocytic lymphohistiocytosis is classified as either primary (genetic) or secondary (acquired) [11,18]. Infections are the most common trigger for secondary HLH, particularly viral (e.g., Epstein–Barr virus), bacterial, and parasitic infections [11]. VL is a recognized, albeit rare, infectious trigger, contributing to the condition often termed “Infection-associated Hemophagocytic Syndrome” (IAHS) [19]. The underlying mechanism involves a defective cytotoxic pathway (T-lymphocytes and NK cells), which fails to eliminate the infected macrophages (which contain *Leishmania* amastigotes) [19]. This failure leads to sustained immune activation, perpetual cytokine release (e.g., Interferon-gamma, Tumor Necrosis Factor-alpha), and the characteristic features of HLH: fever, cytopenias due to suppressed hematopoiesis, and hemophagocytosis (macrophages engulfing blood cells) in the reticuloendothelial organs [20].

Diagnosis of secondary HLH is typically based on fulfilling five out of the eight criteria specified in the revised HLH-2004 protocol or by meeting the H-Score threshold [21]. The key laboratory findings shared by our patients—cytopenia (anaemia and thrombocytopenia), fever, splenomegaly, extremely high ferritin (>2000 ng/mL), and hypertriglyceridemia—strongly align with the diagnostic criteria for HLH [21]. In the diagnostic algorithm for adult HLH, the role of hyperferritinemia is paramount yet nuanced. While the widely utilized HLH-2004 criteria establish a diagnostic threshold of ≥500 ng/mL, this level is frequently surpassed in various adult inflammatory conditions, including bacterial sepsis and advanced liver disease, thereby offering limited specificity [21,22]. However, as ferritin levels increase, its sensitivity and specificity in diagnosing HLH in adults increases [23]. Literature suggests that in adults, a threshold of >2000 ng/mL offers a more reliable trade-off between sensitivity and specificity, while levels exceeding 6000–10,000 ng/mL are considered highly specific for HLH, often reaching a specificity of over 95% [24]. In our reports, the extreme hyperferritinemia (>20,000 ng/mL in Case 2) functioned as a critical diagnostic catalyst. It prompted the immediate pursuit of a bone marrow aspirate to look for hemophagocytosis. The BMA findings confirming hemophagocytosis in both cases provided the final piece of the diagnostic puzzle.

The core therapeutic principle in secondary HLH is the prompt identification and eradication of the underlying trigger [20]. In both of our cases, the administration of liposomal Amphotericin B, the recommended first-line treatment for VL, resulted in significant clinical and laboratory improvement. Case 2 provided a striking example of this principle. The patient was critically ill and met the criteria for HLH, yet treatment was limited solely to the antileishmanial agent. The patient’s spectacular improvement confirmed that eliminating the VL parasite was sufficient to shut down the cytokine storm driving the HLH syndrome. This highlights that in many cases of VL-associated HLH, the HLH component is reversible by treating the primary infection, potentially avoiding the toxicities associated with standard HLH immunosuppressive regimens (e.g., etoposide and dexamethasone). Conversely, Case 1, who presented with more pronounced signs of multi-organ damage, specifically severe coagulopathy and AKI, required the addition of immunoglobulin therapy to manage the severe HLH manifestations. This suggests that patients diagnosed with VL who are critically ill or exhibit multiple organ dysfunctions may benefit from the combined approach, where specific immunomodulatory therapy is temporarily used as a bridge to stabilize the patient while the antileishmanial drug indicates its efficacy. While the HLH-2004 protocol remains the standard for primary HLH, its use in secondary, infection-associated HLH is controversial, particularly when organ failure is present. In Case 1, the presence of severe acute kidney injury (Cr: 4.17 mg/dL) and significant jaundice (Total Bilirubin: 10.08 mg/dL) served as a relative contraindication to the immediate initiation of etoposide, which carries a risk of exacerbated toxicity in patients with renal and hepatic impairment. Consequently, high-dose IVIG was selected as a safer immunomodulatory ‘bridge.’ IVIG may help neutralize pro-inflammatory cytokines and provide passive immunity without the risk of further marrow suppression or organ toxicity, making it a viable option in critically ill patients. This stratified approach is supported by recent clinical data suggesting that while treating the underlying infection is the priority in secondary HLH, patients with high H-scores and impending organ failure may require temporary adjunct immunomodulation to prevent irreversible tissue damage [25]. Early recognition of secondary HLH (sHLH) and combination treatment with IVIG and corticosteroids seem an efficient treatment option with successful outcomes in this life-threatening condition [25].

The incidence of HLH complicating VL was historically considered a rare phenomenon in the pediatric population, with fewer than 50 cases documented in the global literature prior to 2021 [26]. This perceived rarity is supported by data from Bode et al., who reported a 2.1% prevalence of HLH among German VL patients, predominantly within pediatric cohorts [27]. Even in hyper-endemic regions such as India—which historically accounts for the vast majority of global VL cases—the association remained sporadically reported [28]. Similar trends of low prevalence, characterized primarily by isolated case reports, have been observed in Tunisia, Turkey, Sudan, and Iran [29,30,31,32]. However, recent data from Brazil indicate a significantly higher burden. Daher et al. identified HLH in 27.5% (35/127) of pediatric VL patients in the country’s Northeast region [33]. Furthermore, a significant study conducted in the North of Minas Gerais identified a 15.1% prevalence in a cohort of 258 children under ten years of age [19]. These findings underscore the imperative to maintain a high index of clinical suspicion for HLH in VL patients, particularly when the clinical trajectory remains refractory to standard antileishmanial therapy. In contrast, in non-endemic regions, secondary HLH is more frequently precipitated by viral triggers, most notably the Epstein–Barr virus, which is often associated with a more deleterious prognosis compared to parasitic triggers.

The clinical trajectory of HLH exhibits distinct age-related variations and severe multisystemic implications [34]. While pediatric HLH is characterized by an invariably fatal outcome if untreated, adult cohorts may occasionally experience spontaneous remission, though the overall mortality rate remains substantial at approximately 41% [11,35]. Accurate assessment of adult fatality rates is often confounded by the difficulty in distinguishing deaths caused directly by HLH from those resulting from the underlying trigger, such as malignancy or infection [34]. Retrospective data from the HLH-94 protocol demonstrated a 3-year survival rate of 55% in children, which increased to 60–70% among those who successfully underwent bone marrow transplantation (BMT) [36]. The prognosis is notably poorer in malignancy-associated HLH, where median survival is approximately 2.8 months, compared to 10.7 months in non-malignancy-associated cases [34]. Major complications include acute respiratory distress syndrome (ARDS), myocarditis, microangiopathic renal injury, and fatal meningoencephalitis, alongside acute hepatic failure [11,34]. Ultimately, the majority of fatalities are the result of catastrophic hemodynamic collapse, reflecting the devastating and indiscriminate nature of this hyperinflammatory syndrome across all major organ systems [11]. Early recognition, accurate diagnosis, and prompt treatment initiation with liposomal Amphotericin B is vital in cases of HLH secondary to VL in order to alter the course of the disease which could complicated with multiple organ failure, secondary septic shock, hemorrhagic shock, and antimony-related myocarditis [37].

## 4. Conclusions

Although VL-HLH is frequently associated with pediatric cohorts in regions like Brazil, our cases highlight an increasing incidence in older adults with pre-existing comorbidities in the Mediterranean basin. Ultimately, recognizing the diagnostic mimicry of VL-associated HLH is essential to prevent the cytokine-driven tissue damage that leads to fatal outcomes, ensuring that targeted therapy remains the cornerstone of management. Our cases reinforce the necessity of considering VL in the differential diagnosis of fever of unknown origin accompanied by pancytopenia, splenomegaly, and HLH markers (hyperferritinemia, hypertriglyceridemia). The rapid and favorable response to liposomal Amphotericin B confirms that targeted antileishmanial therapy is the cornerstone of managing VL-associated HLH. Treatment for the HLH component should be individualized based on the patient’s severity of illness, reserving intensive immunomodulatory therapies for those with evidence of critical organ failure refractory to the initial antileishmanial regimen.

## Figures and Tables

**Table 1:** Comparison of Key Laboratory Findings at Presentation in Two Adult Patients with Visceral Leishmaniasis (VL)-Associated Hemophagocytic Lymphohistiocytosis (HLH).

*Laboratory* *Findings*	Normal Ranges *	CASE 1			CASE 2		
		Day 0	Day 7	Day 14	Day 0	Day 7	Day 14
*WBC*	4000–10,000/µL	5440	5325	7250	4340	4870	5669
*HB*	13.5–17.5 g/dL (for males)	6.8	8.4	10.2	10.3	11.1	13.1
*HCT*	38.8–50.0% (for males)	20	24.9	29.9	29.6	31.8	36.5
*PLT*	150–450 × 10^3^/L	34,000	67,000	125,000	85,000	99,000	1 = 9 = 39,000
*INR*	0.8–1.2	2.41	1.45	1.06	1.23	1.11	1.02
*PT*	9.4–12.1 s	28	14.2	12.1	14.4	11.9	10.8
*APTT*	25–35 s	51.8	44.2	34.1	61.5	51.9	42.8
*Ur*	7–20 mg/dL	138	79	42	42	25	26
*Cr*	0.6–1.2 mg/dL	4.17	2.67	1.72	0.94	0.75	0.69
*AST*	0–41 U/L	158	102	56	236	194	85
*ALT*	0–40 U/L	60	51	39	124	75	42
*LDH*	140–280 U/L	965	802	560	849	742	523
*Total* *Bilirubin*	0–1.2 mg/dL	10.08	4.56	1.05	1.81	1.23	0.98
*Direct* *Bilirubin*	0–0.2 mg/dL	6.66	3.12	0.6	1.03	0.92	0.51
*ALP*	30–120 U/L	148	125	85	322	302	195
*γ-GT*	7–32 U/L	180	142	101	224	175	129
*CRP*	<0.5 ng/mL	16.81	11.25	5.42	13.76	8.52	3.45
*Ferritin*	30–400 ng/mL	>2000	990	467	>2000	875	326
*Triglycerides*	<150 mg/dL	839	459	251	307	228	194

* Normal values provided by Laboratory of Microbiology of University General Hospital of Alexandroupolis.

## Data Availability

The research data are available after apply to the corresponding author.
